# β-Propeller Blades as Ancestral Peptides in Protein Evolution

**DOI:** 10.1371/journal.pone.0077074

**Published:** 2013-10-15

**Authors:** Klaus O. Kopec, Andrei N. Lupas

**Affiliations:** 1 Department of Protein Evolution, Max-Planck-Institute for Developmental Biology, Tübingen, Baden-Württemberg, Germany; University of Rome, Italy

## Abstract

Proteins of the β-propeller fold are ubiquitous in nature and widely used as structural scaffolds for ligand binding and enzymatic activity. This fold comprises between four and twelve four-stranded β-meanders, the so called blades that are arranged circularly around a central funnel-shaped pore. Despite the large size range of β-propellers, their blades frequently show sequence similarity indicative of a common ancestry and it has been proposed that the majority of β-propellers arose divergently by amplification and diversification of an ancestral blade. Given the structural versatility of β-propellers and the hypothesis that the first folded proteins evolved from a simpler set of peptides, we investigated whether this blade may have given rise to other folds as well. Using sequence comparisons, we identified proteins of four other folds as potential homologs of β-propellers: the luminal domain of inositol-requiring enzyme 1 (IRE1-LD), type II β-prisms, β-pinwheels, and WW domains. Because, with increasing evolutionary distance and decreasing sequence length, the statistical significance of sequence comparisons becomes progressively harder to distinguish from the background of convergent similarities, we complemented our analyses with a new method that evaluates possible homology based on the correlation between sequence and structure similarity. Our results indicate a homologous relationship of IRE1-LD and type II β-prisms with β-propellers, and an analogous one for β-pinwheels and WW domains. Whereas IRE1-LD most likely originated by fold-changing mutations from a fully formed PQQ motif β-propeller, type II β-prisms originated by amplification and differentiation of a single blade, possibly also of the PQQ type. We conclude that both β-propellers and type II β-prisms arose by independent amplification of a blade-sized fragment, which represents a remnant of an ancient peptide world.

## Introduction

The number of possible amino acid sequences available to proteins is tremendous. At the median protein length of 300 residues, there are 20^300^ (∼10^390^) different amino acid combinations; a number so big that life could not possibly have explored all of these sequences to arrive at the current complement of proteins. Instead, it has become evident that the number of proteins observed today is much smaller and that most proteins resemble other proteins. The reason lies in the descent of modern proteins from autonomously folding units called domains. These domains gave rise to new proteins by amplification, recombination, and divergence, and they are thought to have mostly been established at the time of the last common ancestor.

On the structural level, proteins resemble each other even more than on the sequence level. Owing to biophysical constraints, unrelated sequences converged to the same of only ∼1000 folded conformations found in nature. Therefore, structural similarity alone cannot be used to assess whether two proteins have a common origin. The aforementioned vastness of sequence space however makes it unlikely that two sequences converge to a significant similarity and sequence similarity is therefore considered the hallmark of homology.

Even remote homologs often still adopt the same fold, however in some instances homology could be established for proteins of different folds. These homologies were either established by the detection of homologous fold change [1]–[4] or by evidence for shared conserved supersecondary structures [4]–[8]. The latter are assumed to be remnants of an ancient peptide-RNA world [9]–[13].

However, as homologies become more remote and the number of residues that can be compared decreases, it becomes progressively harder to establish statistically significant similarity between sequences over the background. In such cases it would be beneficial to include structural information into the comparisons, because, even though prone to convergence, structures diverge more slowly than sequences. A method to do this was recently introduced in order to establish cases of distant homology [14]. Its rationale is that homologs were almost identical in sequence and structure when they started to diverge from their common ancestor. Over time, these proteins accumulated differences, resulting in progressively lower similarities both in sequence and, more slowly, also in structure. Due to the continuity of this process, we expect to see a positive correlation between sequence and structure similarity for homologous proteins. In contrast, analogs should have varying degrees of structural similarity, mostly independent of sequence similarity, and sequence similarities should generally be low. Sequence and structural similarity scores of analogs are thus expected to be uncorrelated.

It is conceivable that specific local structures might restrict the possible amino acids at one or more positions of the protein, leading to a similar correlation between structure and sequence similarity. A test of this possibility in an evolutionary study of the origins of outer-membrane β-barrels did not uncover such correlation [14], as expected from the observation that domains are multiply convergent at the supersecondary structure level without an accompanying increase in sequence similarity.

In previous studies, we established homology between proteins of different folds based on the analysis of common fragments [4], [5], [8], [14]. These fragments were presumably already found in the last common ancestor of these proteins and were preserved until today even though the proteins themselves underwent fold-changing events. In one of our studies, we found that β-propellers, which adopt folds comprising 4 to 10 repeats of a 4-stranded β-meander called a ‘blade’, can be seen for the most part to have arisen by the independent amplification and diversification of one ancestral blade [15].

The β-strands in each of these blades are named A to D from the N-terminal innermost strand to the C-terminal outermost one [15]. In most β-propellers, β-strands from both the N- and C-terminal regions of the domain constitute the first blade and form a stabilizing velcro closure. Irrespective of the number of blades, β-strands A, B, and C of different blades are usually superimposable with a root-mean-square deviation (RMSD) of below 1 Å even though the insertions between these β-strands vary [15].

Given their versatility in forming β-propellers with different blade numbers, it seemed possible that blades may represent ancient peptides that also gave rise to other folds. In this study we therefore extended our previous efforts by including structural information in the detection of β-propeller homologs. We used the aforementioned method of analyzing sequence similarity as a function of structural similarity to distinguish homology from cases of structure-induced sequence similarity. Here, we show the results of these analyses and report on four potential homologous of β-propeller blades.

## Methods

### Cluster Maps

#### Dataset SCOPβ+

We created the SCOPβ+ dataset by extending the all-β class of SCOP70 1.75 (current release, dated June 2009, clustered at 70% sequence identity), which we chose as a suitable background for distinguishing β-propeller homologs from analogs with similar secondary structure composition (see Results). The extension step was necessary to include potential β-propeller homologs that are not part of the all-β class of SCOP and to include structures not classified in SCOP.

To establish a scaffold for our extension, we first searched the PDB70 database as available on April 5, 2012 (PDB clustered at 70% sequence identity) using HHpred [16], [17], a sensitive remote homology detection method based on the pairwise comparison of Hidden-Markov-Models (HMMs). As query, we used various β-propellers from SCOP and recurrently found matches to 4- to 8-bladed β-propellers (folds b.66-b.70), type II β-prisms (fold b.78), and WW domains (superfamily b.72.1).

The actual extension step started by including all proteins of the all-β class of SCOP70 and extending it by systematically searching PDB70 with all proteins of the aforementioned scaffolding groups. These searches were conducted using the global-alignment mode of HHsearch, the search procedure of HHpred, and matches below 40% probability were discarded. The similarity of some queries led to overlapping matches to the same template protein. We therefore considered all matches to one template in order of decreasing length and kept only those with more than 50% of their residues not already covered by previously accepted matches. In total, the SCOPΒ+ dataset comprises 3223 entries.

For each entry of SCOPβ+ a multiple sequence alignment was computed with the *buildali.pl* script (a modified PSI-BLAST [18] procedure) and *hhmake* was used to convert the alignments to HMMs – both programs are part of the HHpred package. The HMMs of all entries were kept as query HMMs and additionally a single database HMM was created by merging all of them.

### Clustering Procedure

We searched the SCOPβ+ database with each query HMM using HHsearch in global-alignment mode to obtain an all-vs-all matrix of similarity p-values. These p-values were extracted from the result files and converted to a CLANS [19] input file using the *bio.io.hhpred* and *bio.io.clans* modules of CSB, respectively [20]. The cluster map was computed from the input file using the force-directed layouting method implemented in CLANS (attract and repulse value 10) at a p-value threshold of 1e-5 until equilibrium was reached.

### Spurious Connections

**Figure 1:**
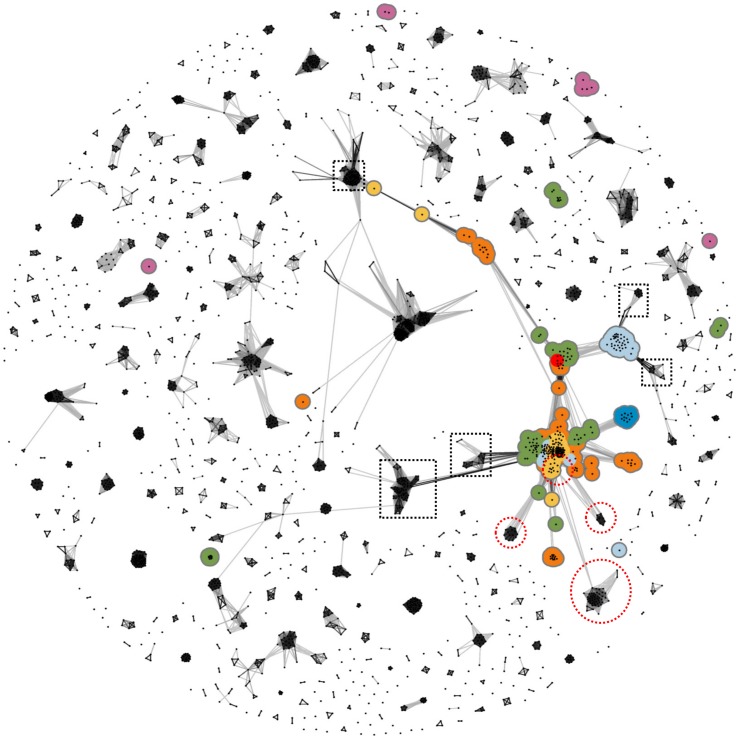
Cluster map of SCOPβ+. β-Propellers are colored by the number of blades (4 = blue, 5 = light blue, 6 = green, 7 = orange, 8 = yellow, 10 = red). Most β-propellers are part of one connected cluster network and they are disconnected from most other clusters. A small number of β-propellers, primarily of viral origin, remain unconnected in sequence space, as discussed previously [15]. Clusters in dashed boxes were omitted in the detailed analysis after manual inspection (see “Spurious connections” in the cluster map section of the Methods). The purple groups are different superfamilies of the β-prism type I fold (b.77), unrelated to the β-prism type II fold discussed in this manuscript (see Fig. 2). The four clusters discussed in this manuscript are in red circles.

We found false-positive connections in the cluster map and removed them after manual verification (dashed boxes in Figure 1). A representative example stems from the extension search with the N-terminal 7-bladed β-propeller in nitrous oxide reductase (SCOP d1fwxa2) as query. This search resulted in matches to a template protein (3HRP) comprising two domains: a 6-bladed β-propeller and an immunoglobulin-like E set domain. Due to a misaligned match, both template domains were covered and instead of the expected β-propeller domain almost the complete protein was included in SCOPβ+. In the cluster map, this protein was located amidst β-propeller proteins due to its β-propeller domain, but is also – and spuriously so – connected to the immunoglobulin domains.

### Correlation of Structural and Sequence Similarity

Dataset. First, we created a template dataset consisting of all single-chain SCOP70 entries as well as the β-pinwheels and the luminal domains of inositol-requiring enzyme 1 (IRE1-LD) proteins. We created HMMs for all 13654 proteins in the dataset as described in the section on cluster map creation.

Next, we chose proteins for a ‘background’ dataset, which contains the SCOP all-β class structures that were neither β-propellers nor considered potential homologs of them, i.e. we excluded β-pinwheels, type II β-prisms, and WW domains. We used this dataset to evaluate which correlation levels are to be expected for structurally similar yet analogous proteins.

Finally, we assembled a query dataset of 583 blade-like structures from all β-propellers (SCOP folds b.66-b.70), type II β-prisms (b.78), and WW domains (superfamily b.72.1) of SCOP70, β-pinwheel fragments, and IRE1-LD fragments. This dataset contains blades and similar β-meanders that we extracted by manual inspection of the structures.

WW domains were restricted to four residues before the first and three residues after the second conserved tryptophan, similar to their Pfam definition (PF00397) [21].

The sequences of β-pinwheels are not continuous when considering β-strands A–D of one blade in structural order. This makes it impossible for TM-align to reasonably align β-pinwheel and β-propeller blades. Thus, we ‘rewired’ the main chain of all β-pinwheel blades by inserting the residues of β-strands B and C (the putative β-hairpin invasion) in between β-strands A and D of their blade. We mapped the positions of the reordered residues to the standard β-pinwheels and computed sequence scores using their HMMs.

Both IRE1-LD structures (PDB 2BE1 and 2HZ6) contain five potential blade homologs, however two of them are not in a β-meander conformation but in a long, extended β-hairpin. We excluded the two elongated instances as the structural alignment score for them would not be meaningful and added the remaining three blade-like fragments to the dataset.

As the full-length proteins of all fragments in the query dataset are in the template dataset, we mapped the query fragments onto them, which allowed us to use the template HMMs for sequence score computations.

#### Correlation calculations

We aligned each query-template pair using TM-align [22] and obtained the query length-normalized TM-score, which we used as our structural similarity score. The TM-score is a value in the interval ]0, 1] where perfectly identical structures have a value of 1 whereas random pairs of structures have a value of ∼0.17 [23], [24]. Sequence similarity scores were calculated by aligning the query and template HMMs according to the TM-align structural alignment using HHalign, the HHsearch scoring procedure. The score HHalign returned was normalized by the number of aligned residues. For the sake of simplicity, we call these sequence similarity scores ‘HHalign-scores’ for the remainder of the manuscript. In the final step, we used SciPy [25] to calculate the correlation between TM- and HHalign-scores for subsets of the query and template datasets.

#### Correlation significance

To assess the statistical significance of each correlation, we assumed a linear dependency between TM- and HHalign-scores. For each set of comparisons (e.g. the set of scores of all comparisons of IRE1-LD queries against PQQ motif β-propeller templates), we did a linear regression (using SciPy) and computed a t-test with the null hypothesis that the slope is zero. In other words, we assessed whether the TM- and HHalign-scores are significantly related. We chose a significance level of 1e-3 and, unless otherwise noted, the correlation values in this manuscript imply significant relationships.

## Results

**Figure 2:**
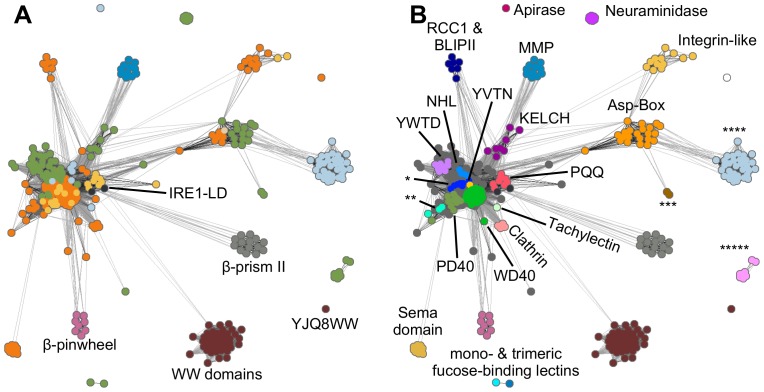
Cluster map of β-propellers and their potential homologs. Dots represent proteins, connections are similarities where darker means more similar. **A)** Potential β-propeller homologs are labeled and structural groups are colored as in Figure 1. The highly divergent WW domain YJQ8WW is annotated as well. **B)** The same cluster map as in A with β-propellers colored and labeled according to motifs and families; names too long to include in the figure are: * = Nitrous oxide reductase N-terminal domain, ** = prolyl oligopeptidase N-terminal domain, *** = Bacteriophage K1F endo-alpha-sialidase, and **** = glycoside hydrolase family 43, ***** = Hemagglutinin-Neuraminidase.

To detect homologs of β-propellers, we clustered the SCOPβ+ dataset based on pairwise sequence similarities. Almost all β-propellers clustered together, as already observed in our previous analysis of the evolution of β-propellers (Fig. 1) [15]. For a detailed inspection, we concentrated on proteins with direct or transitive connections to β-propellers at a p-value cutoff of 1e-5 and omitted all others (Fig. 2A). To annotate groups within this map, we reclustered it at a more stringent cutoff (1e-15), which clearly resolved many groups and allowed us to annotate them by manual inspection. The annotations were transferred to the initial cluster map where the groups remained well defined and resolved, also at the less stringent cutoff used for this map (Fig. 2B).

The cluster map depicts the high degree of interconnectedness between different groups of β-propellers (Fig. 2). The biggest cluster acts as a hub for the connections to the outer clusters and is formed by 5- to 8-bladed propellers of known groups: WD40, KELCH, YWTD, YVTN, NHL [15], PQQ [26], Clathrin [27], and PD40 (Pfam PF07676). The proximity of these different groups in the cluster map indicates close homology, yet the different groups form distinguishable subclusters.

Adjacent to the hub, three β-propeller clusters are formed by the 4-bladed Hemopexin-like domain family (SCOP identifier b.66.1.1), the RCC1/BLIP-II superfamily (b.69.5), and the loosely connected 7-bladed Sema domain superfamily (b.69.12). Also directly connected to the hub is a large cluster formed by the Asp-Box β-propellers, which are mostly 6- and 7-bladed but also contain the only known 10-bladed β-propeller Sortilin [28]. The Asp-Box β-propellers are further tightly connected to proteins of the 5-bladed glycoside hydrolase family 43 and more loosely to two 6-bladed Enterobacteria phage K1F β-propellers and to the Integrin cluster.

Interestingly, we found four groups of proteins in the cluster map that are not β-propellers, yet are connected to them: luminal domains of inositol-requiring enzyme 1 (IRE1-LD), type II β-prisms (BP2), β-pinwheels, and WW domains. These groups vary in the strength of their connections to β-propellers, from the loosely connected WW domains outgroup to the highly connected IRE1-LD.

In the following sections, we report on our investigations of each of the four folds with respect to an origin from an ancestral blade.

### Luminal Domain of Inositol-requiring Kinase 1

IRE1-LD (2BE1 and 2HZ6) is located within the main β-propeller cluster. This domain detects unfolded proteins in the endoplasmic reticulum as part of the unfolded protein response [29] and was predicted to adopt a β-propeller fold due to the detection of four blade-like repeats resembling those of an 8-bladed β-propeller [30]. However, both IRE1-LD structures were found to share a unique fold that consists of a flat anti-parallel β-sheet, formed by β-strands from two monomers as part of their homodimer interface, and α-helices on one side of the β-sheet that form a groove [31], [32]. Further, the fold has two lobes that are described as a distorted β-barrel and a partial β-propeller for the yeast structure, and as two β-barrels for the human one [31], [32].

Due to the striking proximity of IRE1-LD to β-propellers in the cluster map, we investigated this relationship in detail. We ran confirmatory HHpred searches with both IRE1-LD proteins as query against the full PDB70 database. The resulting matches were almost exclusively to β-propellers (yeast IRE1-LD: 252 of 258 matches to β-propellers; human IRE1-LD: 332 of 335) and all other matches had low probabilities. Except for a single low-scoring match, the RBB1NT domain of human retinoblastoma-binding protein 1 (2YRV) at 24% probability, all non-β-propeller matches were to WW domains and type II β-prisms, both proteins described later in this article. Reverse searches with the top-ranked β-propeller matches confirmed the connection to IRE1-LD.

Next, we were interested in whether state-of-the-art repeat detection methods would be able to automatically detect the four blade-like repeats previously found with a semi-automated procedure [30]. We ran the sensitive repeat detection tool HHrepID [33] with the two IRE1-LD sequences as query and both runs detected five repeats. The previously described repeats were the first, third, fourth, and fifth repeat in HHrepID, whereas the second repeat was newly detected. While the first, second, third, and fifth repeat had high probabilities (80%–92%), the probability of the fourth repeat varied between yeast and human IRE1-LD (37% and 89%). However, the sequence segment of this repeat was the same as previously reported and it aligned well to the other repeats.

**Figure 3:**
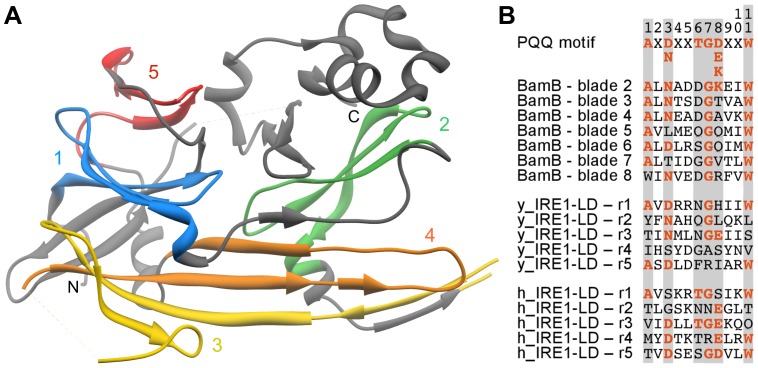
Analysis of IRE1-LD. **A)** Structure of the yeast IRE1-LD monomer with the five repeats detected by HHrepID colored and labeled. **B)** Sequence alignment of the 11-residue PQQ motif in blades 2–8 of BamB (blade 1 is a velcro blade and was omitted for not being continuous in this region) with the corresponding regions of yeast (y) and human (h) IRE1-LD repeats 1–5. At the top is shown the consensus PQQ motif [26]. Conserved motif positions have a gray background and residues adhering to the consensus are highlighted.

Mapping the repeats onto the structure revealed that repeats 1, 2, and 5 are three-stranded β-sheets (Fig. 3). In contrast, repeat 3 contains a long central β-strand and two shorter β-strands that form N- and C-terminal β-β-hairpins with the central one. Repeat 4 comprises two long β-strands that form an elongated β-hairpin. Repeats 1 and 5 constitute the aforementioned partial β-propeller lobe, whereas repeat 2 is part of the putative distorted β-barrel lobe [31]. The elongated repeats 3 and 4 are part of the large β-sheet at the homodimeric interface.

To investigate the structural similarity between IRE1-LD repeats and β-propeller blades, we chose the yeast protein as a representative, as a β-strand of repeat 3 in the human structure is not solved. We superimposed repeats 5 and 1 onto two consecutive blades of the 8-bladed BamB β-propeller (3Q7M), which was the top match in the aforementioned HHpred run. Interestingly, this also superimposed the C-terminal β-hairpin of repeat 3 to the third consecutive blade of the β-propeller, i.e. repeats 5, 1, and 3 are alignable to three consecutive blades. The superimposition aligns the three repeats to the outer blade β-strands, which is peculiar given that strand D is known to be the structurally least conserved one in β-propellers [15]. The newly detected repeat 2 is slightly more distorted than repeats 1 and 5 and therefore did not align as well to β-propeller blades. In a superimposition of repeat 2 and one BamB blade, repeat 3 again comes close to the subsequent β-propeller blade, albeit not as well as when repeats 5 and 1 are used to set the superimposition.

The aforementioned BamB, along with many other top matches of the IRE1-LD HHpred searches, belongs to the PQQ family of β-propellers. These proteins contain an 11 residue motif on β-strands C and D of each blade, which ends with a tryptophan at position 11 (Fig. 3) [26]. The motif comprises two key structural components: (1) residues 6 and 7 of one blade are arranged parallel to the indole ring of Trp11 from the previous blade and (2) the main chain carbonyl of residue 4 is hydrogen-bonded to the Trp11 indole NH group within the same blade [26]. We analyzed IRE1-LD with respect to these two features and found that they are mostly conserved in the structural interactions between repeats 5, 1, and 3. In yeast IRE1-LD, repeat 1 interacts with both structural neighbors, whereas in human IRE1-LD only the interaction between repeats 5 and 1 is seen due to missing density. The more distorted repeat 2, as well as the elongated β-hairpin-like repeat 4 do not show these characteristics. As the conserved residues of PQQ β-propellers are located in β-strands C and D, and play a structural role, it is less surprising that the IRE1-LD repeats align to the outer β-strands and not to the usually well-conserved β-strand A.

**Figure 4:**
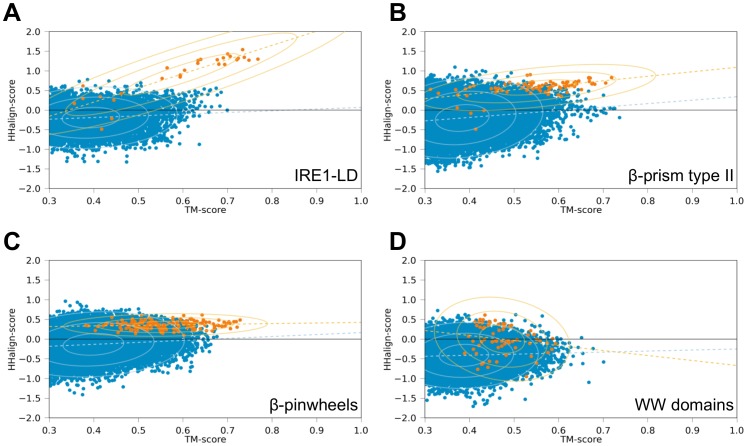
Correlation between structure and sequence similarity in comparisons of the superfamilies analyzed in this study. The comparisons were performed in all cases between a non-propeller fold and the closest β-propeller superfamily, as deduced from Figure 2. The panels show in orange: A) IRE1-LD vs. PQQ, B) β-prism type II vs. PQQ, C) β-pinwheels vs. WD40, and D) WW domains vs. PQQ. In each case, the comparison of the non-propeller fold to a background set of proteins consisting of the SCOP all-β class minus the superfamilies of this study is shown in blue as a reference (see also Methods). The plots represent the structure and sequence similarity of a pair of compared structures as a dot. The linear regression for each group of comparisons is shown as a dashed line, while the ellipses represent one, two, and three standard deviations around the mean.

To further verify these findings, we applied a method that analyzes the correlation between structure and sequence similarity (in the following: sequence-structure correlation; see Methods) (Fig. 4A). We omitted IRE1-LD repeats 3 and 4, as their elongated β-hairpin-like structures make them unsuitable to compute sensible structural alignments to β-propeller blades. The correlation between structure and sequence similarity scores when comparing the IRE1-LD repeats to the background set (see Methods) was 0.11 (median TM- and HHalign-score: 0.38 and −0.18). As the background set is a subset of the SCOP all-β class, a low non-zero correlation was to be expected due to shared β-strand propensity. In comparisons of IRE1-LD to β-propellers with different blade numbers, 8-bladed β-propellers had the highest correlation value (correlation 0.72, TM 0.60, HHalign 0.59). We found that the overall highest correlation was achieved in the comparison to the aforementioned PQQ subset of 8-bladed β-propellers and these comparisons also had remarkably high sequence similarity scores (correlation 0.89, TM 0.63, HHalign 1.17).

Even though IRE1-LD adopts a fold that is globally different from a β-propeller, our analysis indicates that IRE1-LD is closely related to PQQ β-propellers. The antecedent blades are still detectable as repeats even though they only have three β-strands remaining or have changed their conformation. The complexity of the IRE1-LD fold and the five PQQ-like repeats make it unlikely that this fold has arisen by amplification from a single blade. Instead, it is conceivable that a PQQ β-propeller underwent a massive fold change, which was retained due to its emergent usefulness in ER stress sensing.

### Type II β-prism (BP2)

**Figure 5:**
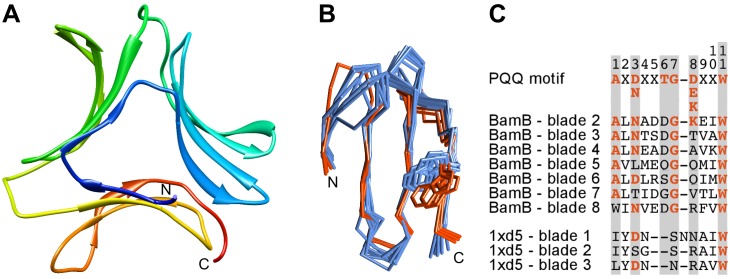
Analysis of type II β-prisms (BP2). **A)** Structure of a BP2 (1XD5), colored blue to red from N- to C-terminus. **B)** Structural alignment of the three β-meanders of a BP2 (1XD5) and the eight blades of BamB (3Q7M) shown as a main chain trace. The 24 aligned residues result in an average RMSD of 1.28 Å. The side-chains of the conserved tryptophan residues in BamB and BP2 are located at the same position but with different orientations. **C)** Sequence alignment derived from the structural alignment in B. See Figure 3 for explanations.

The second group of potential β-propeller homologs in our cluster map are type II β-prisms (BP2, SCOP fold b.78). Proteins with this fold form a superfamily of phylogenetically widespread lectins, referred to as *Galanthus nivalis* agglutinin-related lectins (GNA-related lectins) after the first structure of this fold [34]. The BP2 fold comprises three four-stranded β-meanders that are arranged around and orthogonal to a central pseudo-symmetry axis and are curved towards the center (Fig. 5A). Similar to β-propellers, which circularly permute between one and three β-strands of a terminal blade in order to hydrogen-bond their N- and C-termini and achieve increased stability (velcro closure), BP2 proteins also use velcro closure for their domain organization and dimerization [34], [35]. The sugar-binding motif is located on the outer, concave side of up to three of the β-sheets [36], [37]. Even though sugar binding is their most discussed function, GNA-related lectins also possess 1) anti-tumor, anti-fungal, and anti-viral activity [38], [39], 2) bind the HIV surface glycoprotein GP120 [40], 3) and can be taste modifying [41].

It is important to discriminate BP2 from the type I β-prism (BP1, b.77), which resembles BP2 structurally but has β-strands running parallel to the pseudo-symmetry axis. BP1 proteins also bind carbohydrates with up to three binding sites, and a common origin of BP1 and BP2 has been discussed without clear conclusion [42]. The large distance between BP1 and BP2 proteins in our cluster map (Fig. 1) indicates that even the most sensitive homology detection methods cannot connect them, thus they should be considered analogs.

The BP2 cluster in our cluster map is an outgroup to the 8-bladed PQQ β-propellers, which are found in the central cluster. A multiple-structure alignment of the three β-sheets of a BP2 (1XD5) and the eight blades of a PQQ β-propeller (BamB, 3Q7M) shows that all three BP2 β-sheets align well with PQQ blades (Fig. 5B). Further, a conserved tryptophan in β-strand 4 of the BP2 β-sheets superimposes, with slightly different orientation, onto the conserved tryptophan in position 11 of the PQQ specific motif (see IRE1-LD section). The major difference is a two-residue deletion in the BP2 β-sheets, corresponding to positions 5 and 6 in the PQQ motif (Fig. 5C). BP2 may compensate for the missing stabilizing interaction, which residue 6 provides in PQQ motif blades by coordinating the tryptophan sidechain, through the interaction of its three tryptophan residues in the core of the structure [43]. These differences to the conserved PQQ motif might explain the location of BP2 as an outgroup of PQQ β-propellers.

To verify the presumed homology of BP2 and PQQ β-propellers, we analyzed their sequence-structure correlation (Fig. 4B). The similarity scores for structure and sequence comparisons between BP2 and the background set were low and uncorrelated (correlation 0.16, TM 0.37, HHalign −0.20). In contrast, the comparisons with 8-bladed β-propellers (correlation 0.45, TM 0.53, HHalign 0.48) and their PQQ motif subset (correlation 0.52, TM 0.56, HHalign 0.61) showed similarities indicative of a homologous origin of BP2 from PQQ β-propellers. Sequence searches with single BP2 β-meanders against PDB70 showed that these are more similar to each other than to any β-propeller blade, suggesting that the BP2 repeats were amplified from a single blade of a PQQ β-propeller.

### β-pinwheels

Proteins that adopt the β-pinwheel fold are the third group with connections to β-propellers in our cluster map. They are DNA-binding modules of bacterial type IIA topoisomerases. The first structures with this fold were the C-terminal domains (CTD) of DNA gyrase A (GyrA, 1SUU) and of the topoisomerase IV ParC subunit (1WP5) [44]. DNA gyrase is capable of introducing negative supercoils into DNA, however this function is lost upon removal of either its complete CTD or of a conserved motif therein, the GyrA box [45], [46]. In contrast, topoisomerase IV, which antagonizes DNA gyrase by relaxing supercoiling, remains functional without the CTD but loses specificity for positive supercoiling [44].

**Figure 6:**
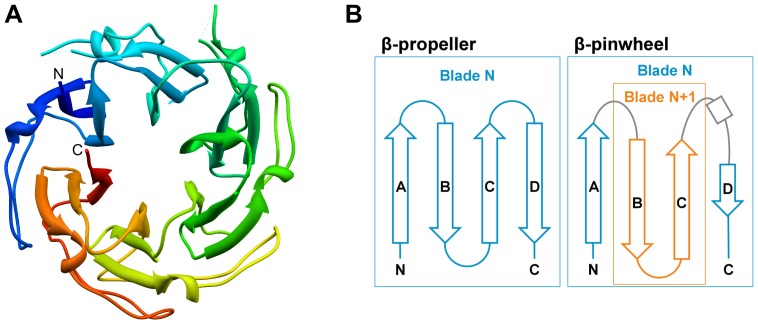
Analysis of β-pinwheels. **A)** Structure of a closed-form β-pinwheel (1SUU). **B)** Topology diagrams of four consecutive β-strands in β-propellers and β-pinwheels. In β-propellers, the four β-strands form a single β-propeller blade. In β-pinwheels, β-strands B and C are part of the next blade, such that the four consecutive β-strands are part of two blades.

Structurally, β-pinwheels resemble β-propellers, with four-stranded β-sheets circularly arranged around a central pore. Yet the folds differ due to a β-hairpin invasion between neighboring β-pinwheel blades (Fig. 6) [47]. Even though they are, strictly speaking, not β-propellers, SCOP classifies them into the 6-bladed β-propeller fold (b.68), where they constitute their own superfamily called “GyrA/ParC C-terminal domain-like” (b.68.10). Interestingly, β-pinwheel structures exist in different variants: completely closed circular forms and C-shaped open forms that can be planar or spiral-shaped. It has been suggested that GyrA always has six blades whereas the number in ParC varies from three to eight and it was hypothesized that ParC evolved from GyrA [48].

In DALI searches [49] for structures similar to β-pinwheels, using the CTD of GyrA and ParC as query, the β-hairpin invasion leads to a clear separation of matches to β-pinwheels (Z-scores >16) and β-propellers (Z-scores <5), which are the top matches besides β-pinwheels. In these searches, the 6-bladed closed β-pinwheels were most similar to 6-bladed β-propellers, whereas the C-shaped forms with five or six blades had 7-bladed β-propellers as top matches.

In these searches, we found six additional β-pinwheel domains (1ZI0, 1ZVU, 1ZVT, 3L6V, 3NO0, 3UC1) and conducted HHpred searches for all eight β-pinwheels against PDB70. We pooled the results into a non-redundant list and, after the self matches, 33 and 3 of the following 40 matches were to 7- and 8-bladed β-propellers, respectively, and only 4 low-scoring matches were to proteins of other folds. The majority of the β-propeller matches were to 7-bladed β-propellers with the WD40 motif, which is in agreement with the cluster map, where β-pinwheels almost exclusively connect to WD40 β-propellers. For confirmatory reverse searches, we used the 10 best β-propeller matches. In all cases, the best β-pinwheel match had a probability >50% and in eight of ten searches >80%. All reverse searches matched multiple β-pinwheels and the matches were interspersed with matches to various β-propeller groups. An earlier study had proposed RCC1 as the group of β-propellers with the highest similarity to β-pinwheels [50], but our analysis indicates only a transitive connection between these groups via the proteins of the main β-propeller cluster, a finding consistent with the previously noted lack of key RCC1 residues in gyrase A [51].

Due to the rather low sequence similarity of β-pinwheels and WD40 β-propellers, which is also evident from their distance in the cluster map, it is not surprising that the WD40 motif-defining tryptophan and aspartate residues are not conserved in β-pinwheels.

To investigate whether the sequence similarity between β-pinwheels and β-propellers could be structure-induced, we again computed sequence-structure correlations (Fig. 4C). Due to the β-hairpin invasion, TM-align is unable to align β-pinwheel and β-propeller blades in a reasonable way; therefore we created artificially reordered β-pinwheel blades (see Methods). The correlation of structure and sequence similarity between the reordered β-pinwheels and the background set was 0.12 (TM 0.39, HHalign −0.13), which is in line with the results for IRE1-LD and BP2. The correlation of scores between the reordered β-pinwheels and the WD40 β-propellers, which were their best sequence matches, was indistinguishable from the background (correlation 0.12, TM 0.56, HHalign 0.37). Both are higher than for the background set, but there is no significant correlation between them, indicating that the sequence similarity may be structure-induced and thus pointing to a convergent origin of WD40 and β-pinwheels (see Methods), as previously proposed [52].

The apparent similarity of β-pinwheels to β-propellers in sequence searches may be due to the two folds being formed by repeats of the same length and secondary structure. This is because the statistical significance of comparisons between repetitive proteins increases with the number of repeats that can be matched, even when the repeats individually have little or no detectable similarity. In this case, searches with single reordered β-pinwheel repeats did not show even low-scoring matches to β-propellers. We therefore conclude that this similarity is not indicative of homology.

### WW Domain

**Figure 7:**
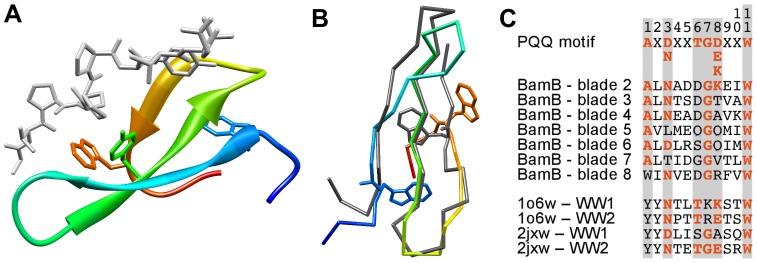
Analysis of WW domains. **A)** Structure of a WW domain (1JMQ_A:13–42) bound to a proline-rich peptide (gray). The side-chains of the conserved tryptophan residues and the binding-site tyrosine are shown. **B)** Superimposition of the first WW domain of PRP40 (1O6W_A:1–29, rainbow coloring) with β-strands B–D of BamB β-propeller blade 2 (3Q7M_A:118–146, dark gray), shown as a main chain trace. The match is gapless and has an RMSD of 1.9 Å over 23 residues. **C)** Sequence alignment of the PQQ motif, BamB blades, and four WW domains. See Figure 3 for explanations.

The fourth group we found connected to β-propellers in our cluster map is the WW domain superfamily (b.72.1). Members of this superfamily adopt a ∼38 residue long fold comprising a curved three-stranded β-meander with two highly conserved tryptophan residues [53]. The N-terminal of these is located in the first β-strand and projects to the convex side of the β-sheet, whereas the C-terminal is in the third β-strand and has its side chain on the concave side. Together with a conserved tyrosine in the central β-strand, the latter forms a binding site for proline-rich motifs (Fig. 7) [54]. WW domains are known to occur in tandems of up to four copies and one reason for this amplification might be to increase binding affinity [55], [56]. Structurally, a WW domain corresponds to three β-strands of one β-propeller blade.

In our cluster map, WW domains are loosely connected to the main β-propeller hub and HHpred searches with single domains often had β-propellers as low-scoring matches, with similar results for the reverse searches. Since, as mentioned for β-pinwheels, the statistical significance of comparisons between repetitive proteins increases with the number of repeats that can be matched, we decided to compare searches with single domains to searches using several domains in tandem.

Searches of single WW domains (1E0L, 1E0N, 1PIN, 1WR4) with HHpred against PDB70 yielded matches to IRE1-LD and several β-propellers, scattered sparsely among other matches and mostly with probabilities below 40% (but occasionally as high as 70%). Although the second conserved tryptophan was in some cases aligned to the conserved tryptophan of PQQ β-propellers and IRE1-LD, many high-scoring matches did not have conserved residues at this position.

Searches of double WW domains (1O6W and 2JXW) showed an increase in number and probabilities of matches to IRE1-LD and β-propellers, particularly to the 8-bladed PQQ β-propellers (up to 93%). Here, two consecutive blades frequently aligned without or with only few gaps to the query WW domains and the conserved C-terminal tryptophan residues in each repeat were aligned.

Searches of quadruple WW domains (gi|73919464∶363–554, gi|2072503∶300–477, gi|73921204∶193–581) confirmed our previous results. Here again, BamB was among the top β-propeller matches (88% probability) and it covered the four WW domains with four consecutive blades, the conserved PQQ motif tryptophan of all four blades being matched to the second WW domain tryptophan.

To assess the structural similarity of WW domains and PQQ motif blades, we compared a double WW domain (1O6W) to its top-matching β-propeller, the 8-bladed BamB, in structure and sequence (3Q7M; Fig. 7B and C). The superimposition had a root-mean square deviation (RMSD) of 1.9 Å over the three β-strands of the WW domain and the alignment was gapless.

As discussed for β-pinwheels, the tandem domains might have elevated scores due to the alignment of multiple consecutive repeats, which in this case might be further enhanced by the repetition of tryptophan at particular sequence intervals. Hence, this finding is not per se indicative of a homologous relationship.

In order to gain more clarity in the issue of homology vs. analogy, we analyzed sequence-structure correlations (Fig. 4D). As in the aforementioned cases, the score correlation between WW domains and the background set was low 0.05 (TM 0.38, HHalign −0.41). To our surprise, neither of the β-propeller groups found in the HHpred analysis had significant correlations with WW domains (correlation against PQQ β-propellers −0.18, TM 0.46, HHalign −0.04). In conjunction with the sequence searches described above, we conclude that the similarity between WW domains and β-propellers is fortuitous and does not reflect common ancestry.

## Discussion

In our search for β-propeller homologs with different folds, we detected four candidate groups: IRE1-LD, BP2, β-pinwheels, and WW domains. These were connected to β-propellers at various levels of statistical significance in sequence comparisons. The question of their evolutionary relationship with β-propellers touches on the problem of distinguishing remote homologs from analogs, a problem that has been discussed for many decades [57], [58]. In this study we have approached this question by complementing detailed, HMM-based sequence comparisons with a recently introduced method that evaluates possible homology based on the correlation between sequence and structure similarity [14]. Our results substantiate a homologous relationship between IRE1-LD, BP2, and β-propellers, but indicate that β-pinwheels and WW domains are most likely of analogous origin.

We have shown previously that β-propellers have arisen for the most part by the independent amplification and diversification of one ancestral blade [15]. A fundamental question in evaluating the evolutionary relationship of IRE1-LD and BP2 to β-propellers is thus whether they also trace their origin to a single blade. In the case of IRE1-LD, the individual repeats are not more similar to each other than to blades of PQQ motif β-propellers and part of the repeats occur in the same geometry. Overall, the IRE1-LD repeats are so similar to PQQ motif blades that they are found in the same sequence cluster, distinct from clusters formed by other β-propellers (Fig. 2). This suggests that IRE1-LD evolved from a PQQ motif β-propeller by a number of mutations that led to a substantial fold change, rather than by amplification of a single PQQ motif blade. We find that the path taken, however, cannot be reconstructed at this time by concatenation of known fold-changing mechanisms [1], [2], [59], since no intermediate forms appear to have survived. We note that the part of the IRE1-LD repeats that can still be related to PQQ motif blades by sequence similarity corresponds to blade β-strands B–D, strand A having been replaced in the process of fold change with heterologous segments of the polypeptide chain.

In the case of BP2, conversely, the high self-similarity of its repeating units and their distinctness from the blades of β-propellers indicate a monophyletic origin from an ancestral blade. While it remains unclear whether the BP2 and β-propeller folds arose concomitantly from the same ancestral blade, or whether BP2 emerged subsequently from the amplification of a β-propeller blade that made itself independent of its parent structure, we note that the particular similarity of BP2 to PQQ motif blades suggests the second scenario, with BP2 arising from the blade of a PQQ β-propeller. In this case, again, the part of BP2 repeats that can be related to PQQ motif blades by sequence similarity corresponds to blade β-strands B–D, strand A being formed by an N-terminal extension that completes each repeat consecutively, constraining the structure to an overall triangular shape (Fig. 5A). It thus seems possible that the BP2 fold arose by amplification of only the three C-terminal β-strands of a PQQ motif blade and that the N-terminal extension providing the fourth strand to each repeat is of heterologous origin. Experimentally, it may be possible to test the viability of this scenario by attempting to complement triple repeats of three-stranded β-meanders derived from the C-terminal part of PQQ motif blades with heterologous sequences in a phage display assay. Nevertheless, whether such a process actually led to the emergence of BP2 remains conjectural at this time, as a higher sequence similarity of BP2 repeats to blade β-strands B–D over other segments of three consecutive β-strands in PQQ β-propellers is not observable.

The homologous relationships highlighted here are exemplary for a problem of current protein classification systems. Due to their tree-like structure and their treatment of structural, i.e. analogous, aspects as the prime mean of differentiation, these systems can only represent homologous connections between proteins that share the same fold. Thereby, fold-spanning homology, as in the cases presented here, cannot be captured. To alleviate this issue, we recently proposed the “metafold” as a new classification level, where homologous proteins can be grouped across different folds [4]. The concept of metafolds can further be applied to bring together proteins that originated from the same ancestral peptide, yet show no global sequence similarity [60]. Once such a systematic grouping of proteins exists, all analogous criteria could be removed from the classification, which would result in a classification by natural descent.

## References

[1] Grishin NV (2001) Fold change in evolution of protein structures. J Struct Biol 134: 167–185. 11551177 10.1006/jsbi.2001.4335

[2] Andreeva A, Murzin AG (2006) Evolution of protein fold in the presence of functional constraints. Curr Opin Struct Biol 16: 399–408. 16650981 10.1016/j.sbi.2006.04.003

[3] Andreeva A, Prlic A, Hubbard TJ, Murzin AG (2007) SISYPHUS–structural alignments for proteins with non-trivial relationships. Nucleic Acids Res 35: D253–259. 17068077 10.1093/nar/gkl746 PMC1635320

[4] Alva V, Koretke KK, Coles M, Lupas AN (2008) Cradle-loop barrels and the concept of metafolds in protein classification by natural descent. Curr Opin Struct Biol 18: 358–365. 18457946 10.1016/j.sbi.2008.02.006

[5] Alva V, Ammelburg M, Soding J, Lupas AN (2007) On the origin of the histone fold. BMC Struct Biol 7: 17. 17391511 10.1186/1472-6807-7-17 PMC1847821

[6] Grishin NV (2001) KH domain: one motif, two folds. Nucleic Acids Res 29: 638–643. 11160884 10.1093/nar/29.3.638 PMC30387

[7] Copley RR, Russell RB, Ponting CP (2001) Sialidase-like Asp-boxes: sequence-similar structures within different protein folds. Protein Sci 10: 285–292. 11266614 10.1110/ps.31901 PMC2373934

[8] Coles M, Hulko M, Djuranovic S, Truffault V, Koretke K, et al (2006) Common evolutionary origin of swapped-hairpin and double-psi beta barrels. Structure 14: 1489–1498. 17027498 10.1016/j.str.2006.08.005

[9] Soding J, Lupas AN (2003) More than the sum of their parts: on the evolution of proteins from peptides. Bioessays 25: 837–846. 12938173 10.1002/bies.10321

[10] Jeffares DC, Poole AM, Penny D (1998) Relics from the RNA world. J Mol Evol 46: 18–36. 9419222 10.1007/pl00006280

[11] Fetrow JS, Godzik A (1998) Function driven protein evolution. A possible proto-protein for the RNA-binding proteins. Pac Symp Biocomput: 485–496. 9697206

[12] Lupas AN, Ponting CP, Russell RB (2001) On the evolution of protein folds: are similar motifs in different protein folds the result of convergence, insertion, or relics of an ancient peptide world? J Struct Biol 134: 191–203. 11551179 10.1006/jsbi.2001.4393

[13] Orgel LE (2004) Prebiotic chemistry and the origin of the RNA world. Crit Rev Biochem Mol Biol 39: 99–123. 15217990 10.1080/10409230490460765

[14] Remmert M, Biegert A, Linke D, Lupas AN, Soding J (2010) Evolution of outer membrane beta-barrels from an ancestral beta beta hairpin. Mol Biol Evol 27: 1348–1358. 20106904 10.1093/molbev/msq017

[15] Chaudhuri I, Soding J, Lupas AN (2008) Evolution of the beta-propeller fold. Proteins 71: 795–803. 17979191 10.1002/prot.21764

[16] Soding J (2005) Protein homology detection by HMM-HMM comparison. Bioinformatics 21: 951–960. 15531603 10.1093/bioinformatics/bti125

[17] Soding J, Biegert A, Lupas AN (2005) The HHpred interactive server for protein homology detection and structure prediction. Nucleic Acids Res 33: W244–248. 15980461 10.1093/nar/gki408 PMC1160169

[18] Altschul SF, Madden TL, Schaffer AA, Zhang J, Zhang Z, et al (1997) Gapped BLAST and PSI-BLAST: a new generation of protein database search programs. Nucleic Acids Res 25: 3389–3402. 9254694 10.1093/nar/25.17.3389 PMC146917

[19] Frickey T, Lupas A (2004) CLANS: a Java application for visualizing protein families based on pairwise similarity. Bioinformatics 20: 3702–3704. 15284097 10.1093/bioinformatics/bth444

[20] Kalev I, Mechelke M, Kopec KO, Holder T, Carstens S, et al (2012) CSB: a Python framework for structural bioinformatics. Bioinformatics 28: 2996–2997. 22942023 10.1093/bioinformatics/bts538

[21] Punta M, Coggill PC, Eberhardt RY, Mistry J, Tate J, et al (2012) The Pfam protein families database. Nucleic Acids Res 40: D290–301. 22127870 10.1093/nar/gkr1065 PMC3245129

[22] Zhang Y, Skolnick J (2005) TM-align: a protein structure alignment algorithm based on the TM-score. Nucleic Acids Res 33: 2302–2309. 15849316 10.1093/nar/gki524 PMC1084323

[23] Xu J, Zhang Y (2010) How significant is a protein structure similarity with TM-score = 0.5? Bioinformatics 26: 889–895. 20164152 10.1093/bioinformatics/btq066 PMC2913670

[24] Zhang Y, Skolnick J (2004) Scoring function for automated assessment of protein structure template quality. Proteins 57: 702–710. 15476259 10.1002/prot.20264

[25] Jones E, Oliphant T, Peterson P (2001 -) SciPy: Open Source Scientific Tools for Python.

[26] Ghosh M, Anthony C, Harlos K, Goodwin MG, Blake C (1995) The refined structure of the quinoprotein methanol dehydrogenase from Methylobacterium extorquens at 1.94 A. Structure. 3: 177–187. 10.1016/s0969-2126(01)00148-4 7735834

[27] ter Haar E, Musacchio A, Harrison SC, Kirchhausen T (1998) Atomic structure of clathrin: a beta propeller terminal domain joins an alpha zigzag linker. Cell 95: 563–573. 9827808 10.1016/s0092-8674(00)81623-2 PMC4428171

[28] Quistgaard EM, Madsen P, Groftehauge MK, Nissen P, Petersen CM, et al (2009) Ligands bind to Sortilin in the tunnel of a ten-bladed beta-propeller domain. Nat Struct Mol Biol 16: 96–98. 19122660 10.1038/nsmb.1543

[29] Ron D, Walter P (2007) Signal integration in the endoplasmic reticulum unfolded protein response. Nat Rev Mol Cell Biol 8: 519–529. 17565364 10.1038/nrm2199

[30] Ponting CP (2000) Proteins of the endoplasmic-reticulum-associated degradation pathway: domain detection and function prediction. Biochem J 351 Pt 2: 527–535. PMC1221390 11023840

[31] Credle JJ, Finer-Moore JS, Papa FR, Stroud RM, Walter P (2005) On the mechanism of sensing unfolded protein in the endoplasmic reticulum. Proc Natl Acad Sci U S A 102: 18773–18784. 16365312 10.1073/pnas.0509487102 PMC1316886

[32] Zhou J, Liu CY, Back SH, Clark RL, Peisach D, et al (2006) The crystal structure of human IRE1 luminal domain reveals a conserved dimerization interface required for activation of the unfolded protein response. Proc Natl Acad Sci U S A 103: 14343–14348. 16973740 10.1073/pnas.0606480103 PMC1566190

[33] Biegert A, Soding J (2008) De novo identification of highly diverged protein repeats by probabilistic consistency. Bioinformatics 24: 807–814. 18245125 10.1093/bioinformatics/btn039

[34] Hester G, Kaku H, Goldstein IJ, Wright CS (1995) Structure of mannose-specific snowdrop (Galanthus nivalis) lectin is representative of a new plant lectin family. Nat Struct Biol 2: 472–479. 7664110 10.1038/nsb0695-472

[35] Chandra NR, Ramachandraiah G, Bachhawat K, Dam TK, Surolia A, et al (1999) Crystal structure of a dimeric mannose-specific agglutinin from garlic: quaternary association and carbohydrate specificity. J Mol Biol 285: 1157–1168. 9887270 10.1006/jmbi.1998.2353

[36] Ramachandraiah G, Chandra NR (2000) Sequence and structural determinants of mannose recognition. Proteins 39: 358–364. 10813817

[37] Shetty KN, Bhat GG, Inamdar SR, Swamy BM, Suguna K (2012) Crystal structure of a beta-prism II lectin from Remusatia vivipara. Glycobiology 22: 56–69. 21788359 10.1093/glycob/cwr100

[38] De Mejia EG, Prisecaru VI (2005) Lectins as bioactive plant proteins: a potential in cancer treatment. Crit Rev Food Sci Nutr 45: 425–445. 16183566 10.1080/10408390591034445

[39] Li Y, Romeis J (2009) Impact of snowdrop lectin (Galanthus nivalis agglutinin; GNA) on adults of the green lacewing, Chrysoperla carnea. J Insect Physiol 55: 135–142. 19041320 10.1016/j.jinsphys.2008.10.015

[40] Hoorelbeke B, Van Damme EJ, Rouge P, Schols D, Van Laethem K, et al (2011) Differences in the mannose oligomer specificities of the closely related lectins from Galanthus nivalis and Zea mays strongly determine their eventual anti-HIV activity. Retrovirology 8: 10. 21314946 10.1186/1742-4690-8-10 PMC3048538

[41] Kurimoto E, Suzuki M, Amemiya E, Yamaguchi Y, Nirasawa S, et al (2007) Curculin exhibits sweet-tasting and taste-modifying activities through its distinct molecular surfaces. J Biol Chem 282: 33252–33256. 17895249 10.1074/jbc.C700174200

[42] Sharma A, Chandran D, Singh DD, Vijayan M (2007) Multiplicity of carbohydrate-binding sites in beta-prism fold lectins: occurrence and possible evolutionary implications. J Biosci 32: 1089–1110. 17954971 10.1007/s12038-007-0111-3

[43] Liu W, Yang N, Ding J, Huang RH, Hu Z, et al (2005) Structural mechanism governing the quaternary organization of monocot mannose-binding lectin revealed by the novel monomeric structure of an orchid lectin. J Biol Chem 280: 14865–14876. 15649901 10.1074/jbc.M411634200

[44] Schoeffler AJ, Berger JM (2008) DNA topoisomerases: harnessing and constraining energy to govern chromosome topology. Q Rev Biophys 41: 41–101. 18755053 10.1017/S003358350800468X

[45] Kramlinger VM, Hiasa H (2006) The “GyrA-box” is required for the ability of DNA gyrase to wrap DNA and catalyze the supercoiling reaction. J Biol Chem 281: 3738–3742. 16332690 10.1074/jbc.M511160200

[46] Kampranis SC, Maxwell A (1996) Conversion of DNA gyrase into a conventional type II topoisomerase. Proc Natl Acad Sci U S A 93: 14416–14421. 8962066 10.1073/pnas.93.25.14416 PMC26147

[47] Hsieh TJ, Farh L, Huang WM, Chan NL (2004) Structure of the topoisomerase IV C-terminal domain: a broken beta-propeller implies a role as geometry facilitator in catalysis. J Biol Chem 279: 55587–55593. 15466871 10.1074/jbc.M408934200

[48] Corbett KD, Schoeffler AJ, Thomsen ND, Berger JM (2005) The structural basis for substrate specificity in DNA topoisomerase IV. J Mol Biol 351: 545–561. 16023670 10.1016/j.jmb.2005.06.029

[49] Holm L, Rosenstrom P (2010) Dali server: conservation mapping in 3D. Nucleic Acids Res 38: W545–549. 20457744 10.1093/nar/gkq366 PMC2896194

[50] Qi Y, Pei J, Grishin NV (2002) C-terminal domain of gyrase A is predicted to have a beta-propeller structure. Proteins 47: 258–264. 11948780 10.1002/prot.10090

[51] Stevens TJ, Paoli M (2008) RCC1-like repeat proteins: a pangenomic, structurally diverse new superfamily of beta-propeller domains. Proteins 70: 378–387. 17680689 10.1002/prot.21521

[52] Corbett KD, Shultzaberger RK, Berger JM (2004) The C-terminal domain of DNA gyrase A adopts a DNA-bending beta-pinwheel fold. Proc Natl Acad Sci U S A 101: 7293–7298. 15123801 10.1073/pnas.0401595101 PMC409912

[53] Bork P, Sudol M (1994) The WW domain: a signalling site in dystrophin? Trends Biochem Sci 19: 531–533. 7846762 10.1016/0968-0004(94)90053-1

[54] Sudol M, Recinos CC, Abraczinskas J, Humbert J, Farooq A (2005) WW or WoW: the WW domains in a union of bliss. IUBMB Life 57: 773–778. 16393779 10.1080/15216540500389039

[55] Hofmann K, Bucher P (1995) The rsp5-domain is shared by proteins of diverse functions. FEBS Lett 358: 153–157. 7828727 10.1016/0014-5793(94)01415-w

[56] Webb C, Upadhyay A, Giuntini F, Eggleston I, Furutani-Seiki M, et al (2011) Structural features and ligand binding properties of tandem WW domains from YAP and TAZ, nuclear effectors of the Hippo pathway. Biochemistry 50: 3300–3309. 21417403 10.1021/bi2001888

[57] Fitch WM (1970) Distinguishing homologous from analogous proteins. Syst Zool 19: 99–113. 5449325

[58] Russell RB, Saqi MA, Sayle RA, Bates PA, Sternberg MJ (1997) Recognition of analogous and homologous protein folds: analysis of sequence and structure conservation. J Mol Biol 269: 423–439. 9199410 10.1006/jmbi.1997.1019

[59] Lupas AN, Koretke KK (2008) Computational Structural Biology: Methods and Applications. Singapore: World Scientific Publishing Company, Incorporated. 792 p.

[60] Alva V, Remmert M, Biegert A, Lupas AN, Soding J (2010) A galaxy of folds. Protein Sci 19: 124–130. 19937658 10.1002/pro.297 PMC2817847

