# Correction: β-Propeller Blades as Ancestral Peptides in Protein Evolution

**DOI:** 10.1371/annotation/fee01544-ff98-4ed2-9dc9-7aea9c5fe828

**Published:** 2014-01-16

**Authors:** Klaus O. Kopec, Andrei N. Lupas

This article was republished on January 14th, 2014 due to errors in the order of Figures 4 through 7 in the original article (which can be viewed here: http://www.plosone.org/corrections/pone.0077074.001.cn.pdf).

